# Randomised In Vitro Study Investigating PEEP‐Stability During Application of CPAP With Binasal Prongs and Face Masks

**DOI:** 10.1111/apa.17589

**Published:** 2025-01-24

**Authors:** Hanna Sterzik, Kriszta Molnar, Anette Stauch, Martin Wald, Christian F. Poets, Bianca Haase

**Affiliations:** ^1^ Department of Neonatology University Children's Hospital of Tuebingen Tuebingen Germany; ^2^ Center for Peadiatric Clinical Studies (CPCS) University Children's Hospital of Tuebingen Tuebingen Germany; ^3^ Department of Neonatology, Division of Pediatrics and Adolescent Medicine Paracelsus Medical University Salzburg Salzburg Austria; ^4^ Department of Diagnostic and Interventional Radiology University Hospital Tuebingen Tuebingen Germany

**Keywords:** CPAP, in vitro, interface, neonate, prongs, resuscitation

## Abstract

**Aim:**

Face masks and binasal prongs are commonly used interfaces for applying continuous positive airway pressure (CPAP) in neonatology. We aimed to assess CPAP stability in a randomised controlled in vitro study.

**Methods:**

In a simulated resuscitation scenario of a 1000‐g preterm infant with respiratory distress, 20 operators (10 with/without neonatology experience) aimed to maintain a CPAP of 5 cmH_2_O as precisely as possible using face masks or binasal prongs in random order. The primary outcome was the minimum‐achieved CPAP at the Y‐piece (P_Y_min). Secondary outcomes included time to target CPAP, CPAP stability and the impact of operator experience.

**Results:**

Binasal prongs enabled more consistent maintenance of the target CPAP of 5 cmH_2_O than face masks (median [IQR]: P_Y_min: binasal prongs, 3.74 cmH_2_O [3.54–3.88]) vs. 3.20 cmH_2_O (2.72–3.73), with no significant deviation from the target CPAP. Target CPAP was achieved significantly faster with face masks (2.89 s [1.67–5.64] vs. 6.49 s [4.76–13.62] [*p* < 0.05]). No significant differences were observed on the basis of operator experience (*p* > 0.05).

**Conclusion:**

Binasal prongs allow more accurate CPAP maintenance than face masks regardless of the operator's experience, although current clinical studies offer limited evidence on the superiority of nasal interfaces compared with that of face masks.

**Abbreviations**
CPAP: continuous positive airway pressure
NALM: Neonatal Active Lung Model
NICU: neonatal intensive care unit
P_Y_
: mean pressure at the Y‐piece
P_Y_max: maximum achieved CPAP at the Y‐piece
P_Y_min: minimum‐achieved CPAP at the Y‐piece

**Summary**

- Continuous positive airway pressure (CPAP) is often used with binasal prongs or face masks in neonatal resuscitation, with potential differences in CPAP stability between these devices.
- On the basis of this in vitro study (simulating a preterm infant during resuscitation), CPAP was kept more stable with binasal prongs, whereas the target CPAP was achieved faster with face masks, both regardless of the operator's experience.
- Further clinical research is needed to validate these in vitro findings.

## 1 Introduction

Continuous positive airway pressure (CPAP) is widely used for respiratory stabilisation [1], particularly in preterm infants [2]. It has been shown to reduce mortality and the incidence of bronchopulmonary dysplasia [3]. Over the past decades, there has been ongoing debate regarding whether face masks or binasal prongs provide superior respiratory support [4]. Although face masks are more commonly used [5, 6], their use may be associated with apnoea or bradycardia [7]. These adverse effects might be related to improper positioning causing leaks, airway obstructions [8] and the activation of the trigeminal nerve [5].

In a pilot study, higher CPAP fluctuations in face masks than in binasal prongs [9] were observed. Therefore, we aimed to evaluate CPAP stability as the primary outcome comparing face masks and binasal prongs. Other critical factors for successful respiratory support, such as the time required to achieve the target CPAP, were also assessed.

## 2 Methods

**FIGURE 1:**
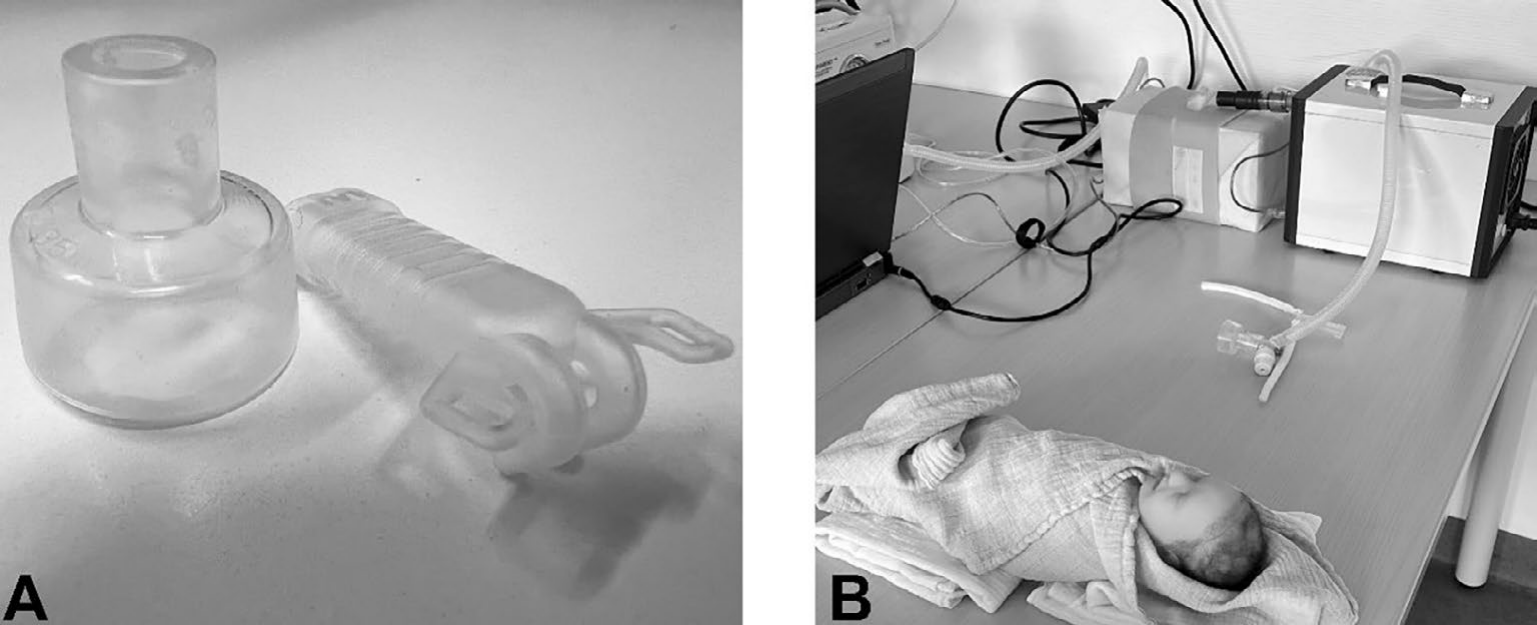
Experimental set‐up, (A) Devices investigated: Left: Face mask (Fisher and Paykel Healthcare, Auckland, New Zealand) 35 mm, right: Binasal Prong (Stephan ‘EasyFlownCPAP’, Gackenbach, Germany), size M (refer Table S1), (B) Experimental set‐up depicting a resuscitation situation: the NALM and 3D‐dummy on the right in the background, with the face mask and t‐piece system positioned in the middle of the image. The premature simulation dummy Paul (Simcharacters, Vienna, Austria) is displayed at the bottom to illustrate the scenario to the test subjects.

For simulating a resuscitation scenario, the operator was positioned at the head of the bed and instructed to administer CPAP at 5 cmH_2_0 to a 1000‐g preterm infant dummy with respiratory distress syndrome. We utilised a Neonatal Active Lung Model (NALM; Dr Schaller Medizintechnik, Dresden, Germany) configured with a respiratory rate of 80/min, tidal volume of 4.5 mL, compliance of 0.4 mL/cmH_2_O and an inspiration time of 30 ms (Table S1). The 3D‐printed nasopharyngeal space dummy was created using a transparent AR‐M2 photopolymer (PFM medical, Koeln, Germany). It was connected to a Perivent/Neopuff (t‐piece) CPAP device (Fisher & Paykel Healthcare, Auckland, New Zealand) using either a 35‐mm neonatal face mask (Fisher & Paykel) or equivalent‐size M nasal prongs (Fritz Stephan GmbH, Gackenbach, Germany) (used devices and experimental set‐up: Figure 1). Apart from the interface used, all other factors were kept constant. The Perivent was calibrated once at the start of the study, with no further adjustments made thereafter. A gas flow of 8 L/min 21% O_2_ (unheated, unhumidified [10]) was employed.

We recruited 20 personnel, consisting of individuals with and without experience in neonatal respiratory support. Experience in neonatology was defined as having worked in a neonatal intensive care unit (NICU) for a minimum of 6 months as either a physician or nursing staff. Each participant was instructed to apply CPAP as consistently as possible using both a face mask and binasal prongs in random order. Data acquisition through the NALM began after a set‐up period of 20 (face mask) 40 (binasal prongs) seconds. During this period, operators aimed to achieve the standard CPAP level of 5 cmH_2_O used in resuscitation [6, 11]. Following this initial phase, simulated breathing was initiated, and participants maintained the CPAP level for an additional 100 s. Afterwards, the simulated breathing was stopped, but the participant continued to apply CPAP for a further 10 s. Throughout the entire experiment, participants received feedback exclusively from the Perivent display. They were allowed to adjust CPAP by repositioning the interface but could not alter the Perivent device settings. During the face mask application, participants faced simulated clinical distractions created by a team member (for details, see Appendix S1).

The NALM TDM file was imported into Excel version 1808 (Microsoft, Washington, USA) using TDM Importer Version 21.3.049496 (National Instruments, Texas, USA). Calculations and statistical analyses were performed using custom scripts in Matlab R2022b (Natick, Massachusetts, USA). A measurement series was discarded and repeated if any of the following conditions were met:

- Test person used not only the display of the Perivent but also the screen of the graphical user interface of the NALM.
- Target CPAP was not achieved during the set‐up time.
- Missing data.

### 2.1 Statistical Analysis

For sample size calculation, the detectable difference was set at 0.7 cmH_2_O, with an observed standard deviation (SD) of 0.48 in preliminary tests. The significance level was set at 0.05, with a power of 80%. This yielded a required sample size of *n* = 18 (for both groups of test persons together: experienced and inexperienced). We decided to test *n* = 20 operators, with 10 experienced and 10 inexperienced ones.

For each interface and participant, 100 breaths were evaluated, which leads to an evaluation of 4000 breaths in total.

The primary outcome measured was the ‘absolute minimum CPAP’, defined as the lowest CPAP value recorded per participant and interface. Secondary outcomes included the ‘absolute maximum CPAP’, ‘time to achieve CPAP’, ‘CPAP deviation beginning vs. end’ and ‘loop spread’. ‘Time to achieve CPAP’ was defined as the duration from the start of data acquisition to the point when a minimum CPAP of 4.75 cmH_2_O (95% of the aimed CPAP) was reached. ‘CPAP deviation beginning vs. end’ was defined as the relative change between the CPAP before the onset of simulated breathing (‘CPAP start’) and after its cessation (‘CPAP end’). ‘CPAP start’ was calculated as the mean pressure at the Y‐piece (P_Y_) 10 s before the initiation of simulated breathing. ‘CPAP end’ was calculated as the mean of all P_Y_ values recorded from the cessation of simulated breathing until the end of data acquisition (approximately 10 s).

**FIGURE 2:**
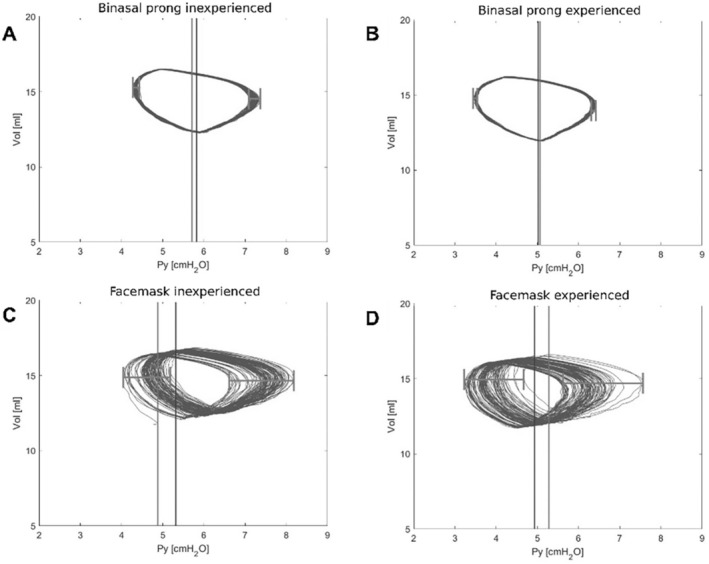
Exemplary Pressure [cmH_2_O] Volume [mL] Loops of two operators visualising all primary and secondary outcomes apart from “time to achieve CPAP”. The light grey vertical line shows the CPAP before starting the simulation breathing; the dark grey vertical line shows the CPAP after stopping it. The horizontal grey line marks the loop spread, which represents the pressure (in)stability of the interface (high loop spread associated with high pressure instability). (A) Inexperienced with binasal prongs, (B) Experienced with binasal prongs, (C) Inexperienced with face mask, (D) Experienced with face mask.

‘Loop spread’ (represented by the horizontal line across the loops in Figure 2) was calculated by identifying the minimum‐achieved CPAP at the Y‐piece (P_Y_min) and the maximum‐achieved CPAP at the Y‐piece (P_Y_max) for each breath. The difference between the highest and lowest P_Y_min and P_Y_max per interface and participant represented the loop spread. All primary and secondary outcomes apart from the ‘time to achieve CPAP’ are visualised by pressure–volume loops (Figure 2).

In addition to analysing the different interfaces, the experience level of the participants was considered a confounding variable.

As the results are not normally distributed (confirmed with the Anderson–Darling test), the Wilcoxon test and the median as central position measure were used. A *p* value < 0.05 was considered statistically significant.

## 3 Results

The analysis comprised 4000 ventilator breaths with 20 different participants and two different interfaces.

Binasal prongs provided a more stable target CPAP of 5 cmH_2_O than face masks.

The median (IQR) of P_Y_min was 3.74 cmH_2_O (3.54–3.88) for binasal prongs (Table S2) compared with 3.20 cmH_2_O (2.72–3.73) for face masks (*p* < 0.05).

For the P_Y_max, the median (IQR) was 6.70 cmH_2_O (6.58–6.80) for binasal prongs and 7.03 cmH_2_O (6.39–7.83) for face masks. No statistically significant difference was found (*p* = 0.38).

With the binasal prongs, the operators achieved a median (IQR) start CPAP of 5.24 cmH_2_O (5.09–5.33), whereas it was 4.87 cmH_2_O (4.23–5.31) with face masks (*p* = 0.06). The CPAP variation from the start to the end of the measurements was 1% using binasal prongs (1%–4%) and 8% (7%–22%) using face masks (*p* < 0.05).

The mean (IQR) pressure deviation from the target CPAP was smaller with binasal prongs (0.31 cmH_2_O [0.16–0.40]) than with the face masks (0.68 cmH_2_O [0.23–1.12]; *p* < 0.05). CPAP stability and accuracy influence the pressure–volume loop spread, which was significantly narrower with binasal prongs (0.15 and 0.19 cmH_2_O on either side of the *p*‐V‐loop) compared to the face mask (0.98 and 1.40 cmH_2_O) (*p* < 0.05) (Figure 2).

**FIGURE 3:**
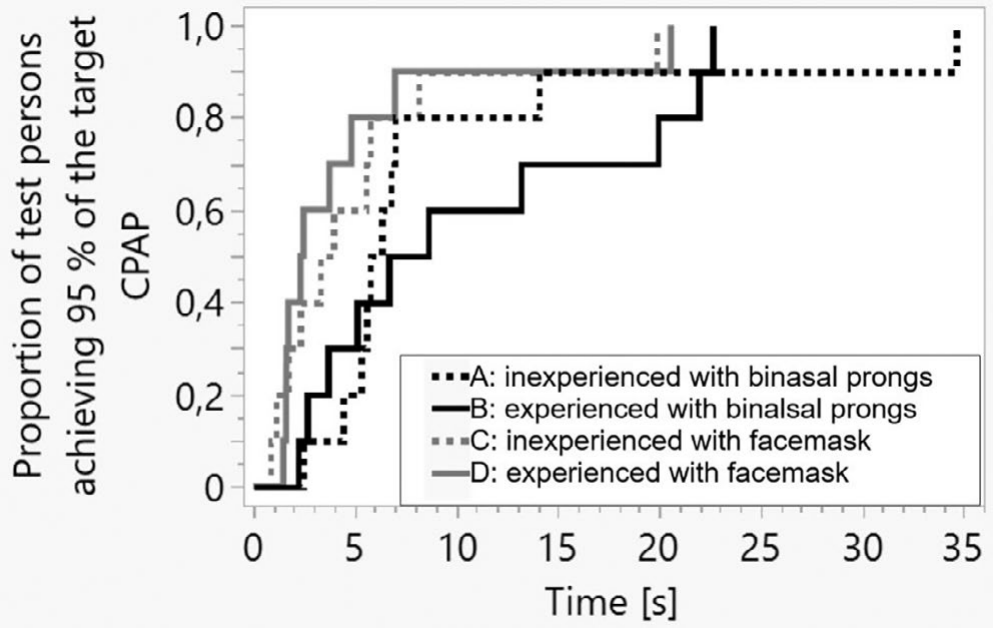
Kaplan–Meier plot for the time it takes to reach 95% of the target CPAP value. The statistical analysis was performed with the Wilcoxon test.

However, achieving 95% of the target CPAP took significantly longer with binasal prongs (6.49 s (4.76–13.62)) than with face masks (2.89 s (1.67–5.64)) (*p* < 0.05) (Figure 3).

No significant differences were observed between experienced and inexperienced operators for either interface (*p* > 0.05), on the basis of the Wilcoxon test for both primary and secondary outcomes. In general, inexperienced operators showed more discrepancies than experienced ones with the face mask than with the prongs (Table S2), but the differences were not statistically significant (shown in interactions in the interaction plot Figure S1).

## 4 Discussion

This analysis tested differences concerning CPAP stability between binasal prongs and face masks. We found that the target CPAP could be applied more accurately with binasal prongs than with face masks. Although different interfaces did not seem to affect the accuracy of the starting CPAP, they did appear to influence maintenance of the target CPAP. This assumption is based on differences in P_Y_min, the wide deviation of the final CPAP from the starting CPAP, the deviation of the mean detected P_Y_ from the target CPAP and loop spread.

On the other hand, face masks allowed a faster CPAP establishment, with 95% of the target CPAP achieved within 3.1 s (Figure 3). This was 50% earlier with face masks than with binasal prongs, where reaching 95% of the target CPAP took longer. Nevertheless, it must be noted that the clinical relevance of this faster CPAP establishment remains uncertain.

The lower CPAP stability observed with face masks may be attributed to the continuous, precise adjustment required by the operator. In contrast, binasal prongs, once accurately positioned, maintained stable CPAP delivery without further adjustment.

Only the P_Y_min demonstrated statistically significant differences between interfaces, whereas the P_Y_max did not. This may lead to the question of whether maximum pressure applied may only partially depend on the operator's applied pressure to the face mask.

In contrast to the pilot study [9], the experience in resuscitation did not seem to have affected any of the factors investigated.

Literature shows little to no evidence of binasal prongs being superior to face masks.

No significant differences in in‐hospital mortality or morbidity between nasal interfaces (prong/tube) and face masks during positive pressure ventilation (PPV) in the delivery room [4, 12] and no differences concerning intubation or mechanical ventilation were found [13]. Although Kuypers et al. [14] detected differences in ventilation modes between binasal prongs, there were no differences in clinical parameters (heart rate and oxygen saturation).

A lower intubation rate in binasal prongs than in face masks was shown by Lamptey et al. [15] and Machumpurath et al. [16] and might be due to the more stable CPAP application (loop spread, Figure 2).

In clinical studies investigating scenarios other than resuscitation, no differences between nasal masks and single nasal prongs in CPAP stability and intermittent hypoxia were shown, suggesting that both non‐invasive interfaces can be interchangeably used [17].

A limitation of our study is its in vitro design. As only the dummy's nostrils were connected to the NALM, a leak through the mouth was not considered. In addition, as the material of the dummy does not reflect the soft skin of a newborn infant and the surface may influence mask application, results may be different in vivo. Furthermore, the child's movements during a resuscitation might affect both the CPAP stability (reflected by the P_Y_min, the P_Y_max, CPAP accuracy and deviation from the beginning to the end, the mean P_Y_ and loop spread) and the duration to achieve the aimed CPAP.

As, for example, the necessary fixation of the prongs with hood and reins was not considered, the setting is, of course, not comparable to the situation in the delivery room, although we tried to create a ‘resuscitation’ scenario for all operators, for example, by introducing external stressors. Finally, we investigated only one kind of binasal prongs and face mask using the Perivent. Our results may not be applicable to all other interfaces used in neonatology as they may differ slightly and thus may influence the parameters we investigated.

Even though there is little to no evidence for the superiority of binasal prongs over face masks during resuscitation, this in vitro study identified a deficiency of face masks in maintaining CPAP levels, which is crucial for successful respiratory support. This fact should be taken into consideration.

## 5 Conclusion

CPAP can be maintained significantly more accurately with binasal prongs than with face masks whereas the target CPAP is reached faster with face masks than with binasal prongs, both regardless of the operator's experience. As an accurate CPAP is crucial for successful respiratory support, this fact should be considered.

## Author Contributions

**Hanna Sterzik:** conceptualization, investigation, writing – original draft, methodology, software, data curation, formal analysis, visualization. **Kriszta Molnar:** conceptualization, writing – review and editing, supervision, data curation, validation. **Anette Stauch:** conceptualization, writing – review and editing, validation. **Martin Wald:** writing – review and editing, validation, methodology, supervision. **Christian F. Poets:** writing – review and editing, supervision, validation, methodology. **Bianca Haase:** conceptualization, investigation, funding acquisition, writing – original draft, methodology, visualization, writing – review and editing, software, formal analysis, supervision.

## Ethics Statement

The trial did not involve patients. The use of the 3D Data for the 3D dummy was approved by the ethics committee of the university of Tübingen under the number 704/2017BO1 for the study: ‘Comparison of nostril sizes of newborn infants with outer diameter of endotracheal tube’.

## Conflicts of Interest

B.H. works as a consultant for PFM Cologne. C.F.P. has received funding from Fritz Stephan GmbH and Loewenstein Medical. The other authors have no conflicts of interest to declare.

## Supporting information


Appendix S1.

## Data Availability

STEP files for 3D dummies, custom‐written MATLAB script, a list of realistic clinical distraction scenarios and detailed result and statistical analysis tables are available upon reasonable request.
