# Supplementary material for: Randomised In Vitro Study Investigating PEEP‐Stability During Application of CPAP With Binasal Prongs and Face Masks
Source: Acta Paediatr. 2025 Jan 24;114(6):1432–6. doi: 10.1111/apa.17589 (PMC12066888; doi:10.1111/apa.17589)
Supplement: Supplementary file 1 — Appendix S1. [file APA-114-1432-s001.docx]

Online Supplement

Title: Randomized controlled in-vitro study investigating the CPAP stability of binasal prongs and face masks

Authors: Hanna Sterzik^1^, Kriszta Molnar^1^, Anette Stauch^2^, Martin Wald^3^, Christian F. Poets^1^, Bianca Haase^1, 4^

^1^ Department of Neonatology, University Children’s Hospital of Tuebingen, Germany

^2^ Center for Peadiatric Clinical Studies (CPCS), University Children’s Hospital of Tuebingen, Germany

^3^ Department of Neonatology, Division of Pediatrics and Adolescent Medicine, Paracelsus Medical University Salzburg, Austria

^4^ Department of Diagnostic and Interventional Radiology, University Hospital Tuebingen, Tuebingen, Germany

Table S1: Settings NALM and interfaces used

| ***Settings NALM*** | |
| --- | --- |
| **Parameter** | **Value** |
| t_ins_ [ms] | 300 |
| f [/min] | 80^1-5^ |
| Aimed V_tid_ [ml] / [ml/kg] | 4.5 / 4.5^1,2,4,5^ |
| Prm [hPa] | 13 |
| Compliance set on the NALM | Cint |
| Compliance [ml / hPa] set on the GUI | 0.4^6-8^ |
| Tube [mm] | 3 |
| Resistance | Ra3 |
| Defined physiologic WOB [mJ / breath] | 1.18^9^ |
| ***Interfaces*** | |
| Prong Stephan „EasyFlow nCPAP“ (Gackenbach, Germany) | size M  ID: 3.1 mm  OD: 4.1 mm |
| Mask Fisher and Paykel Healthcare (Auckland, New Zealand) | Size XS  Diameter 35 mm |

Table S2: Result table

| **Binasal prong**  **all** | **Absolute CPAP min [cmH_2_O]** | **Absolute CPAP max [cmH_2_O]** | **CPAP**  **beginning [cmH_2_O]** | **CPAP end [cmH_2_O]** | **CPAP Deviation beginning vs end in %** | **Deviation of mean pressure to aimed CPAP [cmH_2_O]** | **Loop spread left [cmH_2_O]** | **Loop spread right [cmH_2_O]** | **Time to achieve CPAP [s]** |
| --- | --- | --- | --- | --- | --- | --- | --- | --- | --- |
| **Median** | 3.74 | 6.70 | 5.24 | 5.25 | 1% | 0.31 | 0.15 | 0.19 | 6.49 |
| **1st quartile** | 3.54 | 6.58 | 5.09 | 5.02 | 1% | 0.16 | 0.10 | 0.14 | 4.76 |
| **3rd quartile** | 3.88 | 6.80 | 5.33 | 5.37 | 4% | 0.40 | 0.25 | 0.26 | 13.62 |
|  |  |  |  |  |  |  |  |  |  |
| **Facemask**  **all** | **Absolute CPAP min [cmH_2_O]** | **Absolute CPAP max [cmH_2_O]** | **CPAP beginning [cmH_2_O]** | **CPAP end [cmH_2_O]** | **CPAP Deviation beginning vs end in %** | **Deviation of mean pressure to aimed CPAP [cmH_2_O]** | **Loop spread left [cmH_2_O]** | **Loop spread right [cmH_2_O]** | **Time to achieve CPAP [s]** |
| **Median** | 3.20 | 7.03 | 4.87 | 4.49 | 8% | 0.68 | 0.98 | 1.40 | 2.89 |
| **1st quartile** | 2.72 | 6.39 | 4.23 | 3.70 | 7% | 0.23 | 0.70 | 0.74 | 1.67 |
| **3rd quartile** | 3.73 | 7.83 | 5.31 | 5.18 | 22% | 1.12 | 1.32 | 1.90 | 5.64 |
|  |  |  |  |  |  |  |  |  |  |
| **Binasal prong**  **inexperienced** | **Absolute CPAP min [cmH_2_O]** | **Absolute CPAP max [cmH_2_O]** | **CPAP beginning [cmH_2_O]** | **CPAP end [cmH_2_O]** | **CPAP Deviation beginning vs end in %** | **Deviation of mean pressure to aimed CPAP [cmH_2_O]** | **Loop spread left [cmH_2_O]** | **Loop spread right [cmH_2_O]** | **Time to achieve CPAP [s]** |
| **Median** | 3.72 | 6.72 | 5.20 | 5.25 | 2% | 0.32 | 0.16 | 0.24 | 6,02 |
| **1st quartile** | 3.38 | 6.61 | 5.10 | 5.00 | 1% | 0.21 | 0.11 | 0.17 | 5,30 |
| **3rd quartile** | 3.88 | 7.01 | 5.42 | 5.43 | 5% | 0.55 | 0.25 | 0.33 | 6,95 |
|  |  |  |  |  |  |  |  |  |  |
| **Binasal prong**  **experienced** | **Absolute CPAP min [cmH_2_O]** | **Absolute CPAP max [cmH_2_O]** | **CPAP beginning [cmH_2_O]** | **CPAP end [cmH_2_O]** | **CPAP Deviation beginning vs end in %** | **Deviation of mean pressure to aimed CPAP [cmH_2_O]** | **Loop spread left [cmH_2_O]** | **Loop spread right [cmH_2_O]** | **Time to achieve CPAP [s]** |
| **Median** | 3.83 | 6.69 | 5.24 | 5.26 | 1% | 0.30 | 0.13 | 0.18 | 7.64 |
| **1st quartile** | 3.63 | 6.53 | 5.08 | 5.03 | 1% | 0.11 | 0.10 | 0.12 | 3.65 |
| **3rd quartile** | 3.89 | 6.78 | 5.31 | 5.32 | 2% | 0.36 | 0.24 | 0,21 | 19.91 |
|  |  |  |  |  |  |  |  |  |  |
| **Facemask**  **inexperienced** | **Absolute CPAP min [cmH_2_O]** | **Absolute CPAP max [cmH_2_O]** | **CPAP beginning [cmH_2_O]** | **CPAP end [cmH_2_O]** | **CPAP Deviation beginning vs end in %** | **Deviation of mean pressure to aimed CPAP [cmH_2_O]** | **Loop spread left [cmH_2_O]** | **Loop spread right [cmH_2_O]** | **Time to achieve CPAP [s]** |
| **Median** | 3.40 | 7.10 | 4.87 | 4.21 | 9% | 0.68 | 0.89 | 1.26 | 3,62 |
| **1st quartile** | 2.85 | 6.26 | 4.09 | 3.64 | 6% | 0.23 | 0.66 | 0.52 | 1.70 |
| **3rd quartile** | 3.78 | 7.91 | 5.26 | 5.32 | 36% | 1.13 | 1.19 | 1.70 | 5,76 |
|  |  |  |  |  |  |  |  |  |  |
| **Facemask**  **experienced** | **Absolute CPAP min [cmH_2_O]** | **Absolute CPAP max [cmH_2_O]** | **CPAP beginning [cmH_2_O]** | **CPAP end [cmH_2_O]** | **CPAP Deviation beginning vs end in %** | **Deviation of mean pressure to aimed CPAP [cmH_2_O]** | **Loop spread left [cmH_2_O]** | **Loop spread right [cmH_2_O]** | **Time to achieve CPAP [s]** |
| **Median** | 3.03 | 6.88 | 4.95 | 4.80 | 7% | 0.64 | 1.01 | 1.48 | 2.40 |
| **1st quartile** | 2.70 | 6.43 | 4.38 | 3.83 | 7% | 0.22 | 0.75 | 1.11 | 1.64 |
| **3rd quartile** | 3.57 | 7.76 | 5.40 | 5.04 | 9% | 1.12 | 1.44 | 1.94 | 4.83 |

Figure S1: Interaction Plot


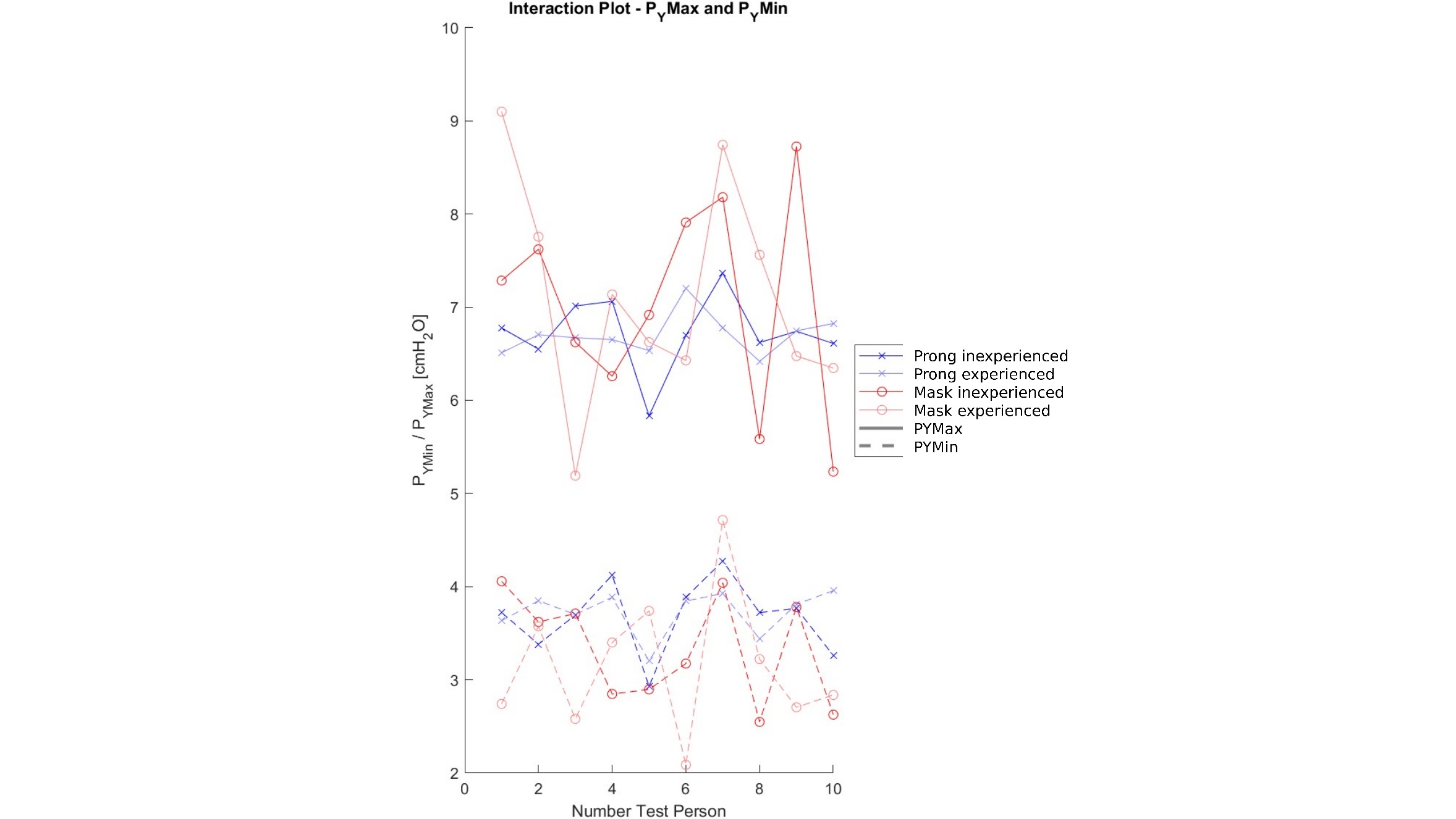


**Table Legends :**

**Table S1:** Overview of the set-up, predefined respiratory parameters set at the NALM (f: frequency, Vtid: tidal volume, tins: inspiration time) and interfaces used (ID: Inner diameter, OD: Outer diameter)

**Table S2:** Measurement result, shown as median, 1^st^ quartile and 3^rd^ quartile of primary (P_Y_min and P_Y_max) and secondary parameters (CPAP onset and end, CPAP deviation at onset vs. end, loop spread left and right, and time to achieve CPAP), A: for all 20 experienced and inexperienced operators with the binasal prongs, B: of all 20 experienced and inexperienced operators with the face mask and subdivided by experience: C: of the 10 inexperienced operators with the binasal prongs, D: of the 10 experienced operators with the binasal prongs, E: of the 10 inexperienced operators with the face mask, F: of the 10 experienced operators with the facemask

**Figure Legends:**

**Figure S1:** Interaction plot of the maximum (continuous line) and minimum (dashed line) pressures [cmH_2_O] measured with facemasks (red) resp. binasal prongs (blue). The lighter colors represent the experienced and the darker colors the inexperienced test persons.

**Reference List**

1. Estol P, Piriz H, Pintos L, Nieto F, Simini F. Assessment of pulmonary dynamics in normal newborns: a pneumotachographic method. *J Perinat Med*. 1988 1988;16(3):183-92. doi:10.1515/jpme.1988.16.3.183

2. Mc IM, Tomlinson ES. The mechanics of breathing in newly born babies. *Thorax*. Mar 1955;10(1):58-61. doi:10.1136/thx.10.1.58

3. Cook CD, Cherry RB, O'Brien D, Karlberg P, Smith CA. Studies of respiratory physiology in the newborn infant. I. Observations on normal premature and full-term infants. *J Clin Invest*. Jul 1955;34(7, Part 1):975-82. doi:10.1172/JCI103165

4. Cook CD, Sutherland JM, Segal S, et al. Studies of Respiratory Physiology in the Newborn Infant. III. Measurements of Mechanics of Respiration1. *Journal of Clinical Investigation*. 1957/03// 1957;36(3):440-448.

5. Mortola JP, Fisher JT, Smith B, Fox G, Weeks S. Dynamics of breathing in infants. *J Appl Physiol Respir Environ Exerc Physiol*. May 1982;52(5):1209-15. doi:10.1152/jappl.1982.52.5.1209

6. Goldsmith LS, Greenspan JS, Rubenstein SD, Wolfson MR, Shaffer TH. Immediate improvement in lung volume after exogenous surfactant: alveolar recruitment versus increased distention. *J Pediatr*. Sep 1991;119(3):424-8. doi:10.1016/s0022-3476(05)82057-8

7. Bhutani VK, Sivieri EM, Abbasi S, Shaffer TH. Evaluation of neonatal pulmonary mechanics and energetics: a two factor least mean square analysis. *Pediatr Pulmonol*. 1988 1988;4(3):150-8. doi:10.1002/ppul.1950040306

8. Nikischin W, Brendel-Muller K, Viemann M, Oppermann H, Schaub J. Improvement in respiratory compliance after surfactant therapy evaluated by a new method. *Pediatr Pulmonol*. Apr 2000;29(4):276-83. doi:10.1002/(sici)1099-0496(200004)29:4<276::aid-ppul7>3.0.co;2-h

9. Abbasi S, Bhutani VK. Pulmonary mechanics and energetics of normal, non-ventilated low birthweight infants. *Pediatr Pulmonol*. 1990 1990;8(2):89-95. doi:10.1002/ppul.1950080206
